# The first Late Triassic Chinese triadophlebiomorphan (Insecta: Odonatoptera): biogeographic implications

**DOI:** 10.1038/s41598-017-01710-7

**Published:** 2017-05-03

**Authors:** Daran Zheng, André Nel, He Wang, Bo Wang, Edmund A. Jarzembowski, Su-Chin Chang, Haichun Zhang

**Affiliations:** 1 State Key Laboratory of Palaeobiology and Stratigraphy, Nanjing Institute of Geology and Palaeontology, Chinese Academy of Sciences, 39 East Beijing Road, Nanjing, 210008 China; 2 Department of Earth Sciences, The University of Hong Kong, Hong Kong, China; 3 Institut de Systématique, Évolution, Biodiversité, ISYEB-UMR 7205-CNRS, MNHN, UPMC, EPHE, Muséum national d’Histoire naturelle, Sorbonne Universités, 57 rue Cuvier, CP 50, Entomologie, F-75005 Paris France; 4 Key Laboratory of Zoological Systematics and Evolution, Institute of Zoology, Chinese Academy of Sciences, 1, Beichen West Road, Beijing, 100101 China; 5 Department of Earth Sciences, The Natural History Museum, London, SW7 5BD UK

## Abstract

The clade Triadophlebiomorpha represents a morphological ‘link’ between the Paleozoic griffenflies (Meganisoptera) and the modern taxa. Nevertheless they are relatively poorly known in the body structures and paleobiogeography. The Triassic dragonfly is extremely rare in China with only one previously recorded. A new family, Sinotriadophlebiidae Zheng, Nel et Zhang fam. nov., for the genus and species *Sinotriadophlebia lini* Zheng, Nel et Zhang gen. et sp. nov., is described from the Upper Triassic Baijiantan Formation of Xinjiang, northwestern China. It is the second Chinese Triassic odonatopteran and the second largest Mesozoic representative of this superorder in China. The discovery provides new information for the clade Triadophlebiomorpha during the Late Triassic and expands its distribution and diversity in Asia. The find reflects a close relationship between the two Triassic entomofaunas from Kyrgyzstan and the Junggar Basin, and provides a Carnian age constraint on the lowermost part of the Baijiantan Formation.

## Introduction

In China, Mesozoic dragonflies have been largely found in Middle Jurassic to Lower Cretaceous strata but are little known from the Triassic although a fragmentary “protodonate” (Zygophlebiidae) has been recently described from the Middle–Upper Triassic Tongchuan Formation of Shaanxi Province, China^1^. In the Junggar Basin of Xinjiang, northwestern China, a few insect fossils have been previously unearthed from Triassic strata^2, 3^. Ten insect orders have been recently recorded from the Lower Jurassic of the Tuziakeneigou outcrop in Karamay City including a damsel-dragonfly *Dorsettia sinica* Zheng *et al*., 2016, which reflects a weak influence of the end-Triassic extinction on damsel-dragonflies or a quick dispersal of damsel-dragonflies during the earliest Jurassic^4^. In addition, four orders of insect fossils have been unearthed from the Upper Triassic Baijiantan Formation of the Shendigou outcrop in Karamay City, somewhat enhancing the distribution and diversity of the insect fauna during the Late Triassic. The clade Discoidalia is divided into two subclades: the Stigmoptera (including the modern Odonata) and Triadophlebiomorpha (Zygophlebiida + Triadophlebiida)^5^. The clade Triadotypomorpha is more widespread than the clade Triadophlebiomorpha as the former occurs in Kyrgyzstan, western Europe (France, Germany and Spain) and Australia^5–7^. All members of the clade Triadophlebiida are known from the Madygen Formation of southwestern Kyrgyzstan^8^.

**Figure 1:**
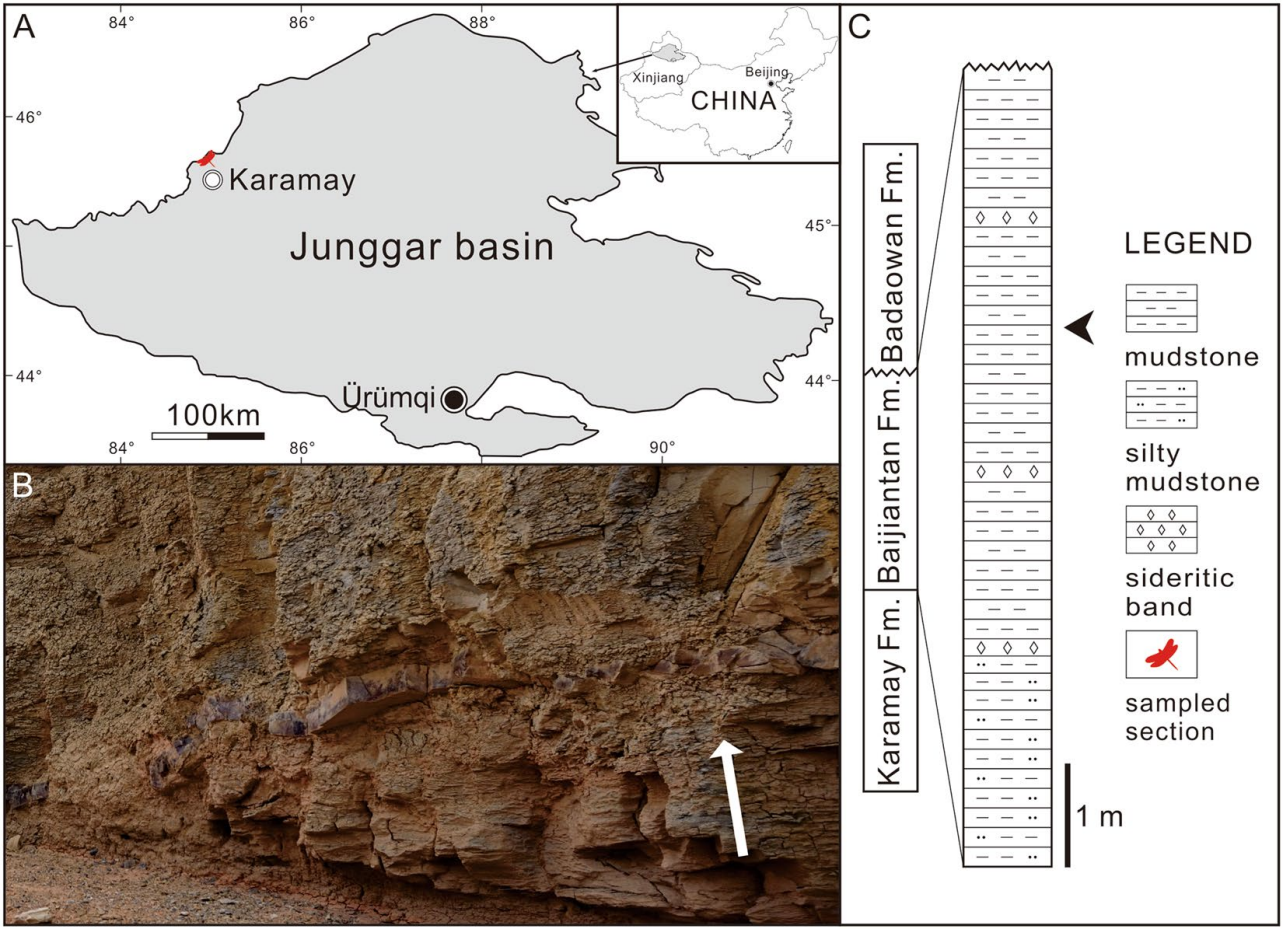
(**A**) Geographic sketch map of the Junggar Basin, Xinjiang, China, showing the locality of the Shendigou outcrop. (**B**) Photograph of the insect-bearing beds of the Baijiantan Formation in the Shendigou outcrop (white arrow length 0.2 m showing the rock orientation). (**C**) Stratigraphic column showing the lithology of the Baijiantan Formation and the position (arrow) of the material collected in the Shendigou outcrop; Fm. = Formation. The map and stratigraphic column are drawn by D.-R.Z. using CorelDRAW X7 (Version number: 17.0.0.491, URL link: http://www.corel.com/cn/).

Here we describe a new family Sinotriadophlebiidae attributable to the clade Triadophlebiida from the Upper Triassic Baijiantan Formation of the Shendigou outcrop, Karamay City, Xinjiang, northwestern China (Fig. 1A,B, 45° 44′ N, 85° 0′ E). The Baijiantan Formation unconformably underlies the Lower Jurassic Badaowan Formation and conformably overlies the Middle–Upper Triassic Karamay Formation in the Shendigou outcrop (Fig. 1C). It consists of grey-white or grey-yellow mudstone and silty mudstone interbedded with sideritic bands, yielding insects, kazacharthrans, fishes, plants and sporopollen and representing a lake facies. It has a total thickness of about 7.7 mm and is considered to be Carnian–Rhaetian (~237–201 Ma) in age in the present paper (see below).

## Material

**Systematic palaeontology**

Order Odonatoptera (*sensu* Brauckmann & Zessin, 1989)

Clade Pandiscoidalia Nel, 2001

Clade Discoidalia Bechly, 1996

Clade Triadophlebiomorpha Pritykina, 1981

Clade Triadophlebiida Bechly, 1996

Family Sinotriadophlebiidae Zheng, Nel *et* Zhang fam. nov.

Genus *Sinotriadophlebia* Zheng, Nel *et* Zhang gen. nov.

Type species: *Sinotriadophlebia lini* Zheng, Nel *et* Zhang sp. nov.

(Figs 2 and 3).

**Figure 2:**
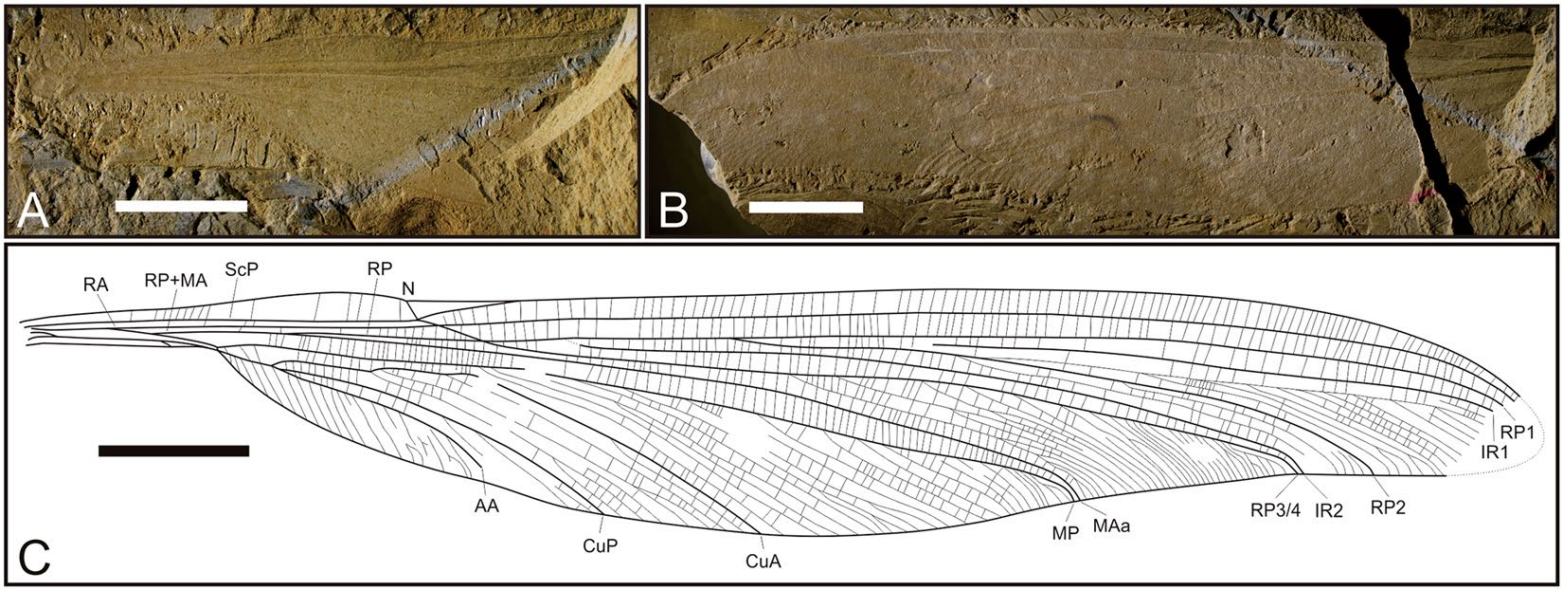
*Sinotriadophlebia lini* Zheng, Nel *&* Zhang gen. et sp. nov. Holotype (NIGP163160); photograph of the basal part (**A**) and distal part (**B**) of the wing, and reconstructed wing drawing (**C**) based on A and B; scale bars = 10 mm. Abbreviations: AA, anterior anal vein; CuA, anterior cubitus; CuP, posterior cubitus; IR, intercalary radial vein; MA, anterior median; MP, posterior median; N, nodus; RA, anterior radius; RP, posterior radius; ScP, posterior subcosta. The line drawing is made by D.-R.Z. using CorelDRAW X7 (Version number: 17.0.0.491, URL link: http://www.corel.com/cn/).

**Figure 3:**
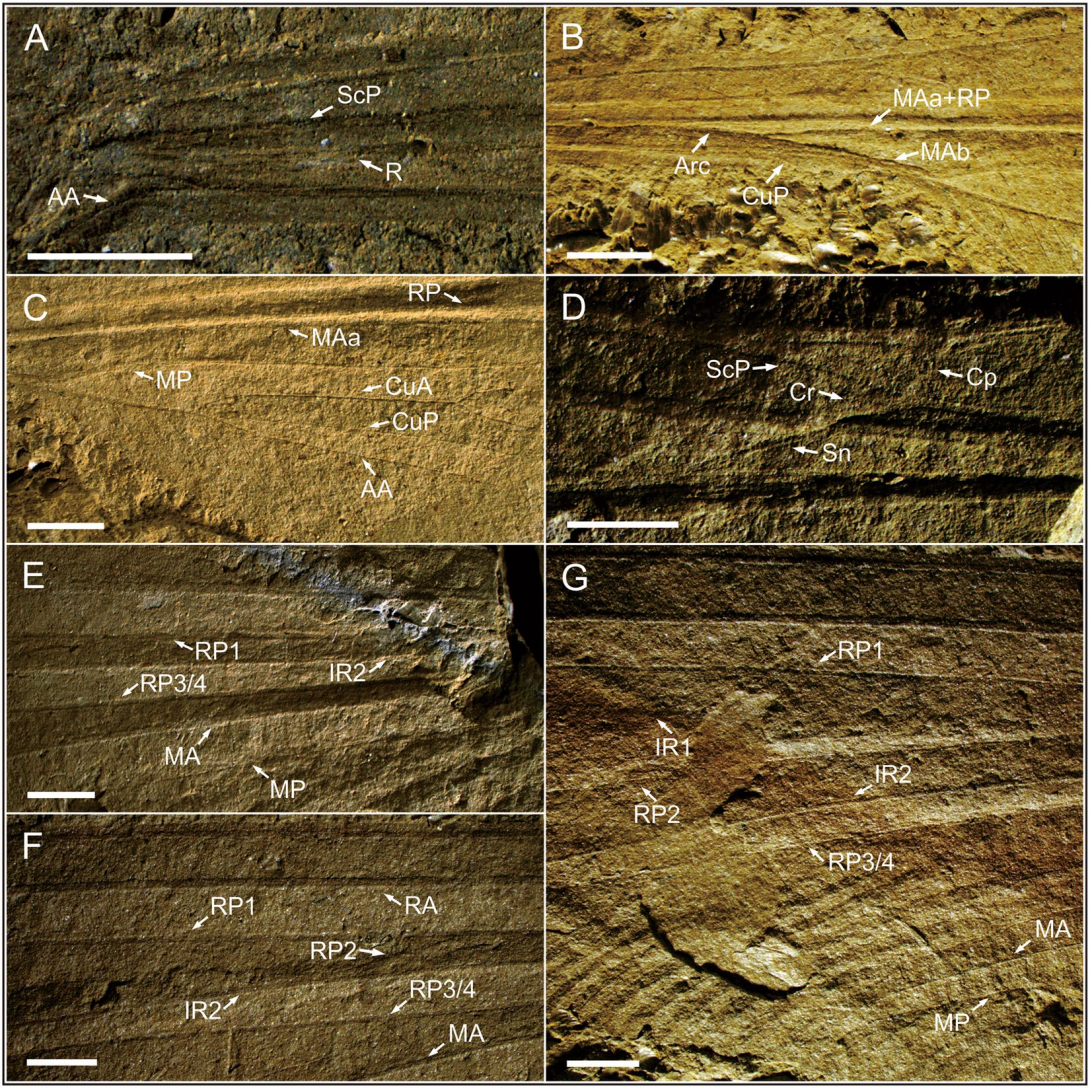
*Sinotriadophlebia lini* Zheng, Nel *&* Zhang gen. et sp. nov. Holotype (NIGP163160); photograph of the wing base (**A**), arculus area (**B**), anal area (**C**), nodal area (**D**), basal area of IR2 (**E**), basal area of RP2 (**F**) and wing apex (**G**); scale bars = 2 mm. Abbreviations: AA, anterior anal vein; Arc, arculus; CP, posterior costa; Cr, nodal crossvein; CuA, anterior cubitus; CuP, posterior cubitus; IR, intercalary radial vein; MA, anterior median; MP, posterior median; R, radius; RA, anterior radius; RP, posterior radius; ScP, posterior subcosta; Sn, subnodal crossvein.

### Diagnosis

AA with numerous parallel posterior branches towards wing margin; ScP crossing N and obliquely reaching wing margin distally with crossveins beneath it; RP and MAa sharing long common basal stem in basal Arc; Sn extremely prolonged and with several crossveins beneath it; CuA with numerous parallel posterior branches.

### Etymology

The genus name is after *Triadophlebia* and Sina, Latin name for China. The species is named after Dr Lin Qibin, a Chinese palaeoentomologist.

### Holotype

Specimen NIGP163160, a slightly damaged wing (part and counterpart), deposited in the Nanjing Institute of Geology and Palaeontology, Chinese Academy of Sciences, Nanjing, China.

### Locality and horizon

Shendigou outcrop, Karamay City, Xinjiang, China; Baijiantan Formation, Late Triassic (Carnian–Rhaetian).

### Description

Wing hyaline, quite elongate, preserved length 99.53 mm, estimated complete length 101–102 mm, width at level of N 10.6 mm, maximum width 16.13 mm; distance from wing base to base of Arc 5.79 mm, and from base of Arc to N 19.95 mm. Nodal structures well preserved (Fig. 3D), showing nodal furrow, slightly posteriorly bent CP, and strongly oblique Cr aligned with oblique Sn (Nel *et al*.^9^: Fig. 5). No obvious primary antenodal crossveins present, probably one (Ax1) located near distal part of MAb; 12 antenodal crossveins present in first row between ScP and C, not aligned with two of second row between ScP and RA. M separating into MA and MP at wing base; MP fused with Cu + AA distally (Fig. 3A). R and MA sharing common stem and separating into RA, RP + MA in Arc. Basal stem RP + MA 3.1 mm long; RP and MAa basally fused for 8.48 mm (Fig. 3C). Long posterior vein MAb present, posteriorly ending on MP + Cu + AA at point where MP separated from Cu + AA (distal end of petiole), distally closing basally open DC. Basal stem of MP + Cu + AA rather long, 10.77 mm in length, divided into MP and Cu + AA at apex of MAb. Basal stem of Cu + AA 3.77 mm long (Fig. 3C). Stem of CuP + AA very short, 0.06 mm long. CuA curved, not parallel to MP with one row of cells basally inbetween, and strongly divergent distally. CuP long, slightly curved, and somewhat parallel to CuA basally. AA with numerous parallel posterior branches, directed towards wing base, with distal part reaching posterior wing margin opposite base of RP3/4. MP slightly curved with several posterior branches. Postdiscoidal area between MAa and MP narrow and converging distally (Fig. 3G). MAa simple. RP3/4 nearly straight, aligned with Sn, with numerous posterior branches between MAa and RP3/4. IR2 simple and probably separated from RP1 at 5.39 mm distal of Sn (Fig. 3E). RP3/4 and IR2 very close near posterior wing margin. RP2 obviously separated from RP1 at 16.43 mm distal of base of RP3/4 (Fig. 3F) with two posterior branches. Base of IR1 not clear; one row of cells between IR1 and RP1; IR1 without posterior branches but a series of secondary longitudinal veins present between IR1 and RP2. No Pt present. Numerous postnodal crossveins present between C and RA, not aligned with numerous postsubnodal crossveins between RA and RP1.

## Discussion

*Sinotriadophlebia* gen. nov. has a highly developed N with a kink in ScP, obvious nodal furrow, aligned Cr and Sn, and a typical odonatoid DC basally open but distally delimited by a characteristically oblique vein MAb between MA and MP. These characters are apomorphies of the Discoidalia Bechly, 1996. It should be noted that Bechly^10^ assigned the two subclades Triadophlebiomorpha Pritykina, 1981 and Triadotypomorpha Bechly, 1996 to the clade Triadophlebioptera Bechly, 1996, considering that Triadotypomorpha also has a typical odonatoid DC closed distally by MAb. However, MAb is in fact absent from *Triadotypus guillaumei* Grauvogel and Laurentiaux, 1952, the type genus of the Triadotypidae Grauvogel and Laurentiaux, 1952 within Triadotypomorpha^11^. In addition, Bechly^10^ put the Piroutetiidae Nel, 1989 in the Triadotypomorpha, a position rejected by Nel *et al*.^5^ who included this family in the Triadophlebiomorpha, and here we restore it in its original position. A further difference between *Sinotriadophlebia* and *Triadotypus* is that MP is simple in the latter but it has numerous posterior branches in the former.

*Sinoriadophlebia* has the following synapomorphies of the Triadophlebiomorpha: petiole very long and of unique shape (not formed by a fusion of CuA + CuP + AA with the anal margin as in other odonates with petiolated wings); MP distinctly curved distal of its origin at the distal angle of DC; and discoidal vein MAb very long and oblique. It also shares a characteristic of this clade in the presence of numerous posterior branches of MP, unlike in the Triadotypomorpha. Within the Triadophlebiomorpha, the affinity of the new genus with the Zygophlebioidea Pritykina, 1981 (*sensu* Bechly, 1996) can be excluded because *Sinoriadophlebia* does not share the peculiar pattern of branching of IR2 and IR1 on RP2 in Zygophlebioidea^8, 10^. *Sinotriadophlebia* also has the following synapomorphies of the Triadophlebiida Bechly, 1996 (Mitophlebiidae Pritykina, 1981 + Triadophlebioidea Pritykina, 1981): CuA + CuP + AA fused with MP and Sn very oblique. The Mitophlebiidae can be excluded from consideration by Sn not being concavely curved in *Sinotriadophlebia*. The Triadophlebioidea comprises Paurophlebiidae Bechly, 1996 and Triadophlebiidae Pritykina, 1981. The absence of the fusion of ScP with RA from the base to N in *Sinotriadophlebia* excludes affinity with Paurophlebiidae and Triadophlebiidae can also be excluded by the following differences: RP3/4 unforked in *Sinotriadophlebia* but forked in Triadophlebiidae; many antesubnodal crossveins present in the former but only one in the latter; and vestige of stem of media not suppressed or completely fused with the hind margin in the former. *Sinoriadophlebia*, however, shares with Triadophlebiidae the secondary fusion of the common stem of MP + Cu + AA with the hind margin of the wing.

**Figure 4:**
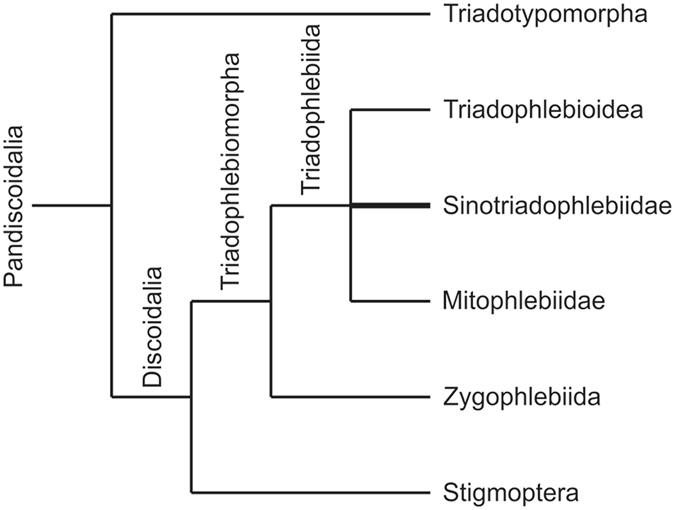
Hypothetical position of Sinotriadophlebiidae in Pandiscoidalia. The line drawing is made by D.-R.Z. using CorelDRAW X7 (Version number: 17.0.0.491, URL link: http://www.corel.com/cn/).

In conclusion, *Sinotriadophlebia* can be attributed to neither the Mitophlebiidae nor the Triadophlebioidea (Fig. 4). A new family is proposed based on the following combined differences: large size with wing length reaching up to 100 mm (within the Triadophlebiida, all species are less than 80 mm except *Neritophlebia longa* Pritykina, 1981 being 120–125 mm in length); distal part of ScP crossing N very obliquely with crossveins beneath it; CuA with numerous parallel posterior branches (a character also present in *Triadotypus*, but more developed than in *Sinotriadophlebia*); RP and MAa fused into a long basal stem (a character also present in *Triadotypus*, but not in all other Triadophlebiida); Sn extremely oblique and very elongate, with several crossveins beneath it (a character shared with the Neritophlebiinae).

*Sinotriadophlebia lini* is the second largest Mesozoic odonatopteran from China, being slightly smaller than the damsel-dragonfly *Hsiufua chaoi* (107.6 mm wing for forewing length) from the Middle-Upper Jurassic of Inner Mongolia^12^. In addition, the Triassic *Iverya averyi* Béthoux & Beattie, 2010 is currently considered within Triadotypidae, showing combined characters of Triadotypidae and Triadophlebiomorpha. It shares the characters present in the Triadophlebiomorpha but not in *Triadotypus*: oblique MAb, well separated RP and MAa, and long petiole. However, it also shares with *Triadotypus* a CuA with numerous posterior branches and a simple MP, unlike in Triadophlebiomorpha^7^. The discovery of much better preserved specimens is necessary to determine its exact position.

**Figure 5:**
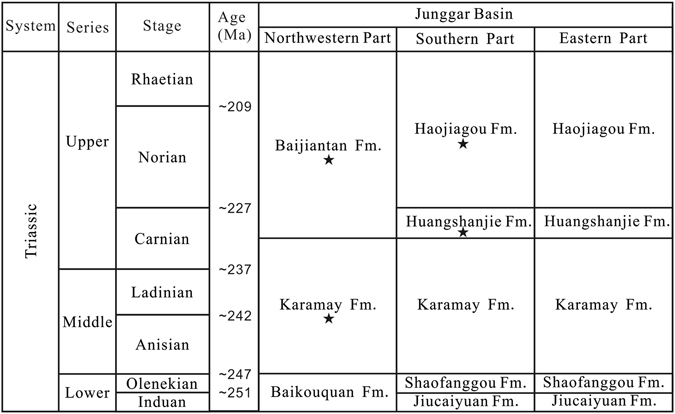
Correlation of the Triassic strata in the Junggar Basin (★star showing the formations yielding insect fossils; Fm. = Formation). The correlation table is made by D.-R.Z. using CorelDRAW X7 (Version number: 17.0.0.491, URL link: http://www.corel.com/cn/).

Numerous fossils including insects, kazacharthrans, fishes, plants and sporopollen have been unearthed from the Baijiantan Formation of the Shendigou outcrop in Xinjiang^13, 14^. The sporopollen assemblage in the Baijiantan Formation is mostly inherited from that of the upper part of the Karamay Formation^13^ and the megaspore assemblage resembles that of the Upper Triassic Haojiagou Formation in the southern Junggar Basin^15^, all indicating a Late Triassic age. The Baijiantan Formation is found in the northwestern Junggar Basin and can be correlated with the Huangshanjie-Haojiagou formations in the southern and eastern basin (Fig. 5). The Huangshanjie and Haojiagou formations are considered to be Carnian^16^ and Norian–Rhaetian in age^17, 18^, separately. Therefore, the age of the Baijiantan Formation is probably Carnian–Rhaetian.

The clade Triadophlebiida were previously only recorded from the Madygen Formation of southwestern Kyrgyzstan^8^. The age of the Madygen Formation is considered to be Ladinian–Carnian^19–21^. The discovery of *Sinotriadophlebia* therefore points to a Carnian age for the lowermost part of the Baijiantan Formation. The relatively short distance between the Madygen fossil site and northwestern Xinjiang suggests a close relationship between the two insect faunas.

## Methods

The specimen was examined dry under a Nikon SMZ1000 stereomicroscope. Photographs were taken using a Canon 5D digital camera and the line drawings were prepared from photographs using image-editing software (CorelDraw X7 and Adobe Photoshop CS6). The specimen is housed in the Nanjing Institute of Geology and Palaeontology, Chinese Academy of Sciences (NIGPAS).

The nomenclature of the odonatopteran wing venation used in this paper is based on the interpretations of Riek^22^ and Riek & Kukalová-Peck^23^, as modified by Nel *et al*.^24^ and Bechly^10, 25^. Vein abbreviations are as follows: AA, anterior anal vein; Ax, primary antenodal crossvein; Arc, arculus; C, costa; CP, posterior costa; Cr, nodal crossvein; CuA, anterior cubitus; CuP, posterior cubitus; DC, discoidal cell; IR, intercalary radial vein; MA, anterior median; MP, posterior median; N, nodus; R, radius; RA, anterior radius; RP, posterior radius; ScP, posterior subcosta; Sn, subnodal crossvein.

The higher classification of Triadophlebiida, as well as family and generic characters followed in the present work, is based on the phylogenetic system proposed by Bechly^25^ and modified by Nel *et al*.^5^.
